# Rapid detection and specific identification of offals within minced beef samples utilising ambient mass spectrometry

**DOI:** 10.1038/s41598-019-42796-5

**Published:** 2019-04-18

**Authors:** Connor Black, Olivier P. Chevallier, Kevin M. Cooper, Simon A. Haughey, Julia Balog, Zoltan Takats, Christopher T. Elliott, Christophe Cavin

**Affiliations:** 1 Institute for Global Food Security, Advanced ASSET Centre, School of Biological Sciences, Queen’s University Belfast, 18-30 Malone Road, Belfast, United Kingdom; 2 Mass Spectrometry Core Technology Unit, Queen’s University Belfast, Belfast, United Kingdom; 3 Waters Research Centre, 7 Zahony Street, Budapest, Hungary; 4 Imperial College London, South Kensington Campus, Sir Alexander Fleming Building, London, United Kingdom; 5 Nestlé Research, Vers-chez-les Blanc, CH-1000 Lausanne 26, Switzerland

**Keywords:** Mass spectrometry, Statistics, Scientific data

## Abstract

The morphological transformation of beef tissues after various processing treatments facilitates the addition of cheap offal products. Undetectable to the naked eye, analytical techniques are required to identify such scenarios within minced and processed products. DNA methodologies are ill-equipped to detect adulteration of offal cuts from the same species and vibrational spectroscopic studies, although rapid and non-destructive, have proved inconclusive as to whether the specific adulterant can be identified. For the first time we present a mass spectrometric approach employing an ambient ionisation process to eliminate sample preparation and provide near-instantaneous results. Rapid evaporative ionisation mass spectrometry (REIMS) was used to assess its capabilities of detecting minced beef adulteration with beef brain, heart, kidney, large intestine and liver tissues and chemometric analysis enabled unique or significant markers to be identified. The adulteration levels detected with the REIMS technology when analysing raw adulterated beef burgers were; brain (5%); heart (1–10%); kidney (1–5%); large intestine (1–10%) and liver (5–10%). For boiled adulterated samples; brain (5–10%); heart (1–10%); kidney (1–5%); large intestine (1–10%) and liver (5–10%). REIMS allows rapid and specific identification of offal cuts within adulterated beef burgers and could provide a paradigm shift across many authenticity applications.

## Introduction

Minced and processed meat products are purchased in most parts of the world by consumers and therefore, constitute a significant proportion of the product ranges of meat companies. With the global processed meat market valued at $714 billion in 2017 and expected to reach $1567 billion by 2022^1^, the potential for economic gains through adulteration is tantalising for criminals and is likely to be heavily exploited. The morphological characteristics of meat tissues are drastically altered, if not totally destroyed as a result of numerous processing treatments. Thus, for consumers it is very difficult, if not impossible to identify adulteration when purchasing such products. Although not a new phenomenon, the 2013 European horsemeat scandal very publicly highlighted how extensive such adulteration operations can be^2^. Unfortunately, such scandals continue to be reported as demonstrated within a recent United Kingdom (UK) based study in which more than a fifth of 665 samples analysed contained non-declared meat species^3^. The substitution of one species with a cheaper alternative species is the most exploited and well known form of meat fraud, however, there are additional ways to achieve fraud in the meat trade including: country of origin, sex, cut, breed, age, processing or treatment and non-meat ingredient additions^4^.

Offal cuts are defined as the internal organs and entrails of a butchered animal. Although considered a delicacy in certain parts of the world, the European Union (EU) Commission Directive 2001/101/ EC introduced a generic labelling scheme for meat which restricts products to skeletal tissue and maximum limits of natural fat and connective tissue^5^. The presence of non-meat offal cuts must be declared on product labels. However, due to significant pricing differences compared with skeletal tissue, the addition of low cost offal cuts to processed meat products is common practice, particularly beef.

DNA sequencing is a highly efficient and accurate analytical platform which has advanced in reducing the time required to obtain identifications (30 minutes excluding sample preparation time)^6^. However, its limitation of only differentiating between tissue samples from different biological species makes it totally ill-equipped to detect offal adulteration within species. Several vibrational spectroscopic studies (Fourier transformed infrared (FT-IR), near-infrared (NIR) and Raman) have demonstrated their ability to detect offal adulteration of meat products very rapidly. Although rapid and non-destructive, few have concluded the capability in identifying which specific offal is present within an adulterated minced beef sample^7–9^. Elemental analysis using laser induced breakdown spectroscopy (LIBS) has also been conducted to detect offal adulteration^10^.

Tandem chromatography-mass spectrometry techniques have been applied extensively towards meat fraud analysis yet^11,12^, the detection of offal cuts within meat samples has not been investigated. This is perhaps an indictment of the time that is required to both prepare and analyse samples compared to other analytical platforms. However, the increasing use of ambient mass spectrometry (AMS) techniques towards food fraud analysis suggests a potential change to the status quo^12,13^. As well as providing rapid and accurate results, the combined use of high resolution mass spectrometry (HRMS) and chemometric analysis enables the potential identification of unique or significant metabolites, an aspect which eludes spectroscopic and elemental composition studies.

For the first time we present a mass spectrometry approach to rapidly detect the adulteration of raw and boiled minced beef products with several beef offal cuts (brain, heart, kidney, large intestine and liver) using rapid evaporative ionisation mass spectrometry (REIMS). This innovative technology enables simultaneous acquisition and classification of samples without the need for any form of sample preparation using a monopolar blade, bipolar forceps or infrared (IR) laser. Initially invented and developed for medical surgical applications^14^, the technology has since been applied to microorganism and bacteria analysis^15–17^, and more recently shown to be extremely proficient at analysing multiple aspects of food fraud, including fish and meat speciation^18–21^.

## Results and discussion

The application of AMS techniques towards the analysis of food fraud and foodomics studies in general has increased in recent years^12,13^. Yet, the study of minced beef adulteration with offal cuts lacks investigation from all mass spectrometric platforms. Qualitative profiling of beef muscle and potential beef offal adulterants was undertaken using the REIMS technology in conjunction with a high-resolution quadrupole time-of-flight (QTof) mass spectrometer. This untargeted approach coupled with chemometric analysis (principal component analysis (PCA), an unsupervised technique, linear discriminant analysis (LDA) and orthogonal partial least squares-discriminant analysis (OPLS-DA), both supervised techniques) enabled the raw spectrometric data to be analysed extensively with no specific variables targeted. As a result, a variety of chemometric models could be generated by altering parameters such as mass range, binning size, scaling parameters (unit variance, pareto, etc.) and number of components.

### Separation of beef muscle and offal tissues

**Figure 1:**
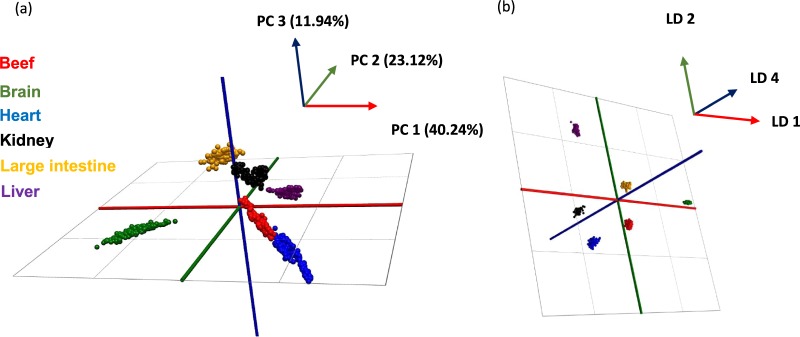
(**a**) PCA score plot and (**b**) LDA plot of REIMS-QTof MS spectral data (m/z 600–950) obtained from beef muscle, brain, heart, kidney, large intestine and liver tissues in negative ionisation and sensitivity mode. Each data point represents a single cut within the experiment of which there were 889 in total. 50 PCA components, 5 LDA components and a mass bin size of 0.5 Da were used. R^2^ = 0.964 and Q^2^ = 0.948 values were obtained for the PCA score plot.

The unsupervised PCA model (Fig. 1a) demonstrated clear separation between beef muscle, brain, heart, kidney, large intestine and liver tissues suggesting sufficient variation within the spectrometric mass range m/z 600–950. Likewise, R^2^ and Q^2^ values of 0.964 and 0.948 respectively suggest successful separation. This is in accordance with previous REIMS studies where glycerophospholipids in the mass range m/z 600–950 have predominately been identified as the main point of discrimination^14–18,20,22^. The beef muscle and heart tissue clusters were in close proximity of one another in the PCA plot which is not to be unexpected as both muscle skeletal and heart cardiac tissue are striated muscles. On the other hand, large intestine is a smooth muscle which does not have the same straited structure whilst brain is a mixture of grey and white matter made up of neurons, dendrites and axons respectively. Likewise, kidney is a complex mixture of inner and outer cortexes which does not contain straited muscle. The supervised LDA score plot (Fig. 1b) also provided separation with six tight clusters. Additional potential offal adulterants (rib bone, rib cartilage, nuchal ligament, paddywhack, fetlock skin, knee joint and stomach lining) were analysed, however, due to ionisation and/or reproducibility issues they were excluded from the model building processes. Attempts to generate further PCA-LDA models focused on an alternative lower mass range (m/z 200–450), where the spectrometric features were dominated by intact fatty acids, and a higher mass range (m/z 950–1200) were also made. However, such was the lack of spectrometric variation within the two ranges that it was not feasible to produce chemometric models which sufficiently separated the six classes. Thus, these models would not have been suitable to be used as a base point to detect offal adulteration in minced burger samples as discussed later.

### Real-time adulteration of beef burgers

**Figure 2:**
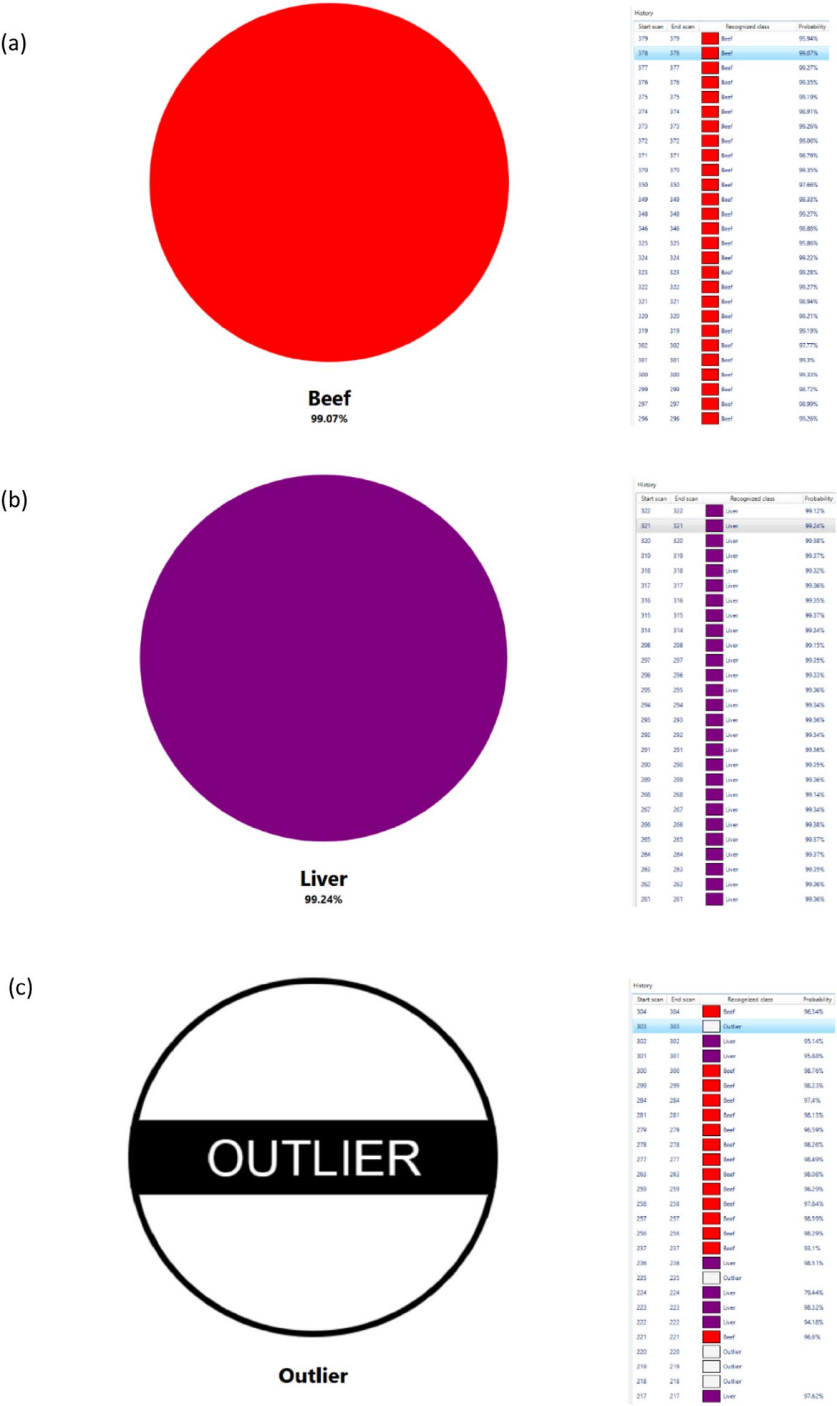
Real-time recognition results obtained using the REIMS technology in conjunction with the recognition software. The figures show the classifications obtained from (**a**) a pure beef sample, (**b**) a pure liver sample and (**c**) an adulterated beef burger with liver at 10% adulteration. The beef and liver samples analysed in figures ‘a’ and ‘b’ were used to prepare the adulterated burger in figure ‘c’. All samples were not present in the original chemometric training model and therefore, considered ‘blind’. The 10% adulterated burger produced beef (red), liver (purple) and outlier (white) classifications with an outlier being a result of the electrosurgical monopolar blade analysing a ‘hybrid’ beef-liver spectrum at a specific scan time which situated outside the 20σ standard deviation range of pure beef and liver.

Analytical techniques capable of delivering reliable, accurate and preferably rapid identifications to ensure the integrity and safety of products are required by food companies. Utilising the PCA-LDA models (Fig. 1), REIMS in conjunction with a recognition software adheres to such requirements. Identifications for the adulterated beef burgers were assigned near-instantaneously (≈ 2 seconds) for each sample cut. The adulteration levels detected for each adulterant were; brain (5%); heart (1–10%); kidney (1–5%); large intestine (1–10%) and liver (5–10%) with a full summary of the findings outlined in Supplementary S1. Specific adulterant and outlier identifications were assigned most frequently for burgers which had been adulterated at the higher levels (20–10%). Importantly, no false positive classifications were assigned. To ensure the reliability of the recognition results, pure samples of the beef muscle and offal samples used to prepare the adulterated beef burgers were subjected to the same method of analysis. No false positive or outlier classifications were observed (Fig. 2a,b).

Outlier classifications were not assigned in recent REIMS studies focused on surgical applications or fish fillet fraud, excluding the analysis of fish species not present within the original training model^20,23^. This is because there are clear interface boundaries between normal and cancerous tissues whilst fish fillets are 100% pure samples which have not been processed or minced. Within this study adulterated burgers were prepared through a vigorous blending process to mimic real-life scenarios. Therefore, outliers were a result of homogenous mixtures of beef and adulterant at a specific scan time. In effect, a ‘hybrid’ spectrum which did not situate within the 20σ standard deviation range for either pure beef or adulterant. Burgers adulterated with heart and, surprisingly, large intestines were the only offal not to provide any outlier classifications. Such are the similarities between beef and heart muscle, hence their proximity within the PCA score plot, that their ‘hybrid’ spectra fell within the 20σ standard deviation for either class. Thus, the percentage composition of the two tissues determined the assigned classification. Contrarily, adulteration with tissues that are significantly different to that of beef, such as brain and liver, produced numerous outlier signals (Fig. 2c). In fact, two of the four burger samples adulterated with brain identified no beef classifications for samples adulterated at 20%. The lack of outlier classifications for burgers that had been adulterated with large intestines was unexpected and may have been a result of poor homogeneity resulting in no ‘hybrid’ spectra. However, there is no explanation as to why only the large intestine adulterated burgers would exhibit such issues. Alternatively, it may be that beef and large intestine samples have the same set of significant peaks, like that of the heart samples, thus one ‘overrules’ the other one resulting in either a beef or large intestine classification.

Two significant observations manifested from the recognition classifications. Firstly, within seconds of analysing a sample the REIMS technology and recognition software (i) identified that a minced beef sample had been adulterated and (ii) confirmed which specific offal adulterant was present, excluding some of the brain adulterated samples. As discussed previously, the latter is an aspect which has eluded all spectroscopic studies excluding that conducted by *Hu et al*.^24^. Secondly, several meat fraud studies, the majority of which are not associated with offal adulteration, have demonstrated that adulteration can be successfully detected at levels < 1%^25–27^. The presence of another meat species or non-meat product at levels as low as 1% is a result of accidental cross contamination and not the deliberate act of food fraud. Such levels cannot be associated with criminal activity as this would not result in any meaningful economic gains. Instead, levels upwards of 15–20% would fall under their purview. Therefore, the adulteration levels detected within this study for each offal adulterant, regardless of outlier classifications, demonstrate that the REIMS technology is more than sufficient for detecting such adulteration.

### Candidate biomarkers

Utilising high resolution mass spectrometric platforms enables vast amounts of data to be extracted through chemometrics. The ability to identify unique or significant markers which can subsequently be transferred to a targeted tandem mass spectrometry (MS/MS) method is of huge importance for metabolomic studies. Such workflows have already been implemented within food analysis, a most recent example being the adulteration of dried oregano products^28,29^.

The identification of the most significant metabolites within the REIMS dataset was undertaken by removing ions with a VIP < 1 leaving 188 ions in which to build individual OPLS-DA models of each tissue type against the other five tissues (statistical parameters – Table 1) and then S-plots. To be considered for REIMS MS/MS analysis an ion had to exhibit significant contribution to the model and appropriate reliability thus, S-plot cut-off values of p[1] (x ≥ 0.03) and p(corr) (x ≥ 0.5) were employed, akin to previous metabolomic studies^19,20^. Table 2 lists the ions chosen and their putatively annotated identifications after conducting REIMS MS/MS analysis. As no chromatographic separation is associated with the REIMS technology and therefore, not applied before the ion fragmentation/identification, a diverse selection of isobaric and isomeric lipid species was observed for each ion. Ceramides (Cer), diacylglycerols (DG), glucosylceramides (GluCer), monogalactosyldiacylglycerols (MGDG), phosphatidic acids (PA), phosphatidylcholines (PC), phosphatidylethanolamines (PE) and phosphatidylglycerols (PG) were the assigned lipid species.

**Table 1:** The statistical parameters observed for each individual OPLS-DA model generated based upon spectrometric data obtained using REIMS-QTof MS in negative ionisation and sensitivity mode. The table identifies the number of latent and orthogonal components used for each model and their subsequent R^2^, Q^2^ and RMSECV values. Individual models were generated excluding ions with a VIP < 1.

Model	Latent component	Orthogonal component	R^2^ (cum)	Q^2^ (cum)	RMSECV
Brain v other classes	1	1	0.985	0.985	0.0476
Heart v other classes	1	5	0.969	0.968	0.0689
Kidney v other classes	1	3	0.964	0.963	0.0681
Large intestine v other classes	1	3	0.968	0.968	0.0655
Liver v other classes	1	1	0.975	0.973	0.0581

**Table 2:** List of ions identified for REIMS MS/MS analysis based upon the S-plot p[1] and S-plot p(corr) values of each ion which identify the contribution of each ion to the variance of the observations and their reliability respectively. Additionally, the ∆ ppm of the accurate mass compared to the true mass of the assigned identifications is stated.

Marker	Ion bin category (Da)	Accurate mass (Da)	S-plot p[1] value	S-plot p(corr) value	∆ ppm	Lipid identification(s)	Adduct(s)
BRN 1	600.75	600.5116	0.319	0.953	0	DG (18:0/18:0) (d5)	[M-H_2_O-H]^−^
					1	Cer (d18:0/18:1)	[M + Cl]^−^
						Cer (d18:1/18:0)	[M + Cl]^−^
BRN 2	628.75	628.5427	0.144	0.907	2	Cer (d18:1/20:0)	[M + Cl]^−^
BRN 3	682.75	682.5913	0.180	0.903	0	Cer (d18:1/24:1)	[M + Cl]^−^
BRN 4	735.25	735.4758	0.171	0.938	2	PA (18:1/18:1)	[M + Cl]^−^
						PA (18:2/18:0)	[M + Cl]^−^
BRN 5	844.75	844.6450	0.157	0.906	1	GluCer (d18:1/24:1)	[M + Cl]^−^
HRT 1	669.25	669.4842	0.179	0.879	—	*N/A*	*N/A*
HRT 2	681.25	681.4861	0.312	0.852	0	PA (O-16:0/20:4)	[M-H]^−^
						PA (P-18:0/18:3)	[M-H]^−^
						PA (P-18:1/18:2)	[M-H]^−^
						PA (18:1/18:1)	[M-H_2_O-H]^−^
						PA (18:2/18:0)	[M-H_2_O-H]^−^
					2	PA (P-18:0/16:0)	[M + Na-2H]^−^
HRT 3	766.75	766.5408	0.262	0.938	2	PC (20:4/16:0)	[M-CH_3_-H]^−^
						PE (20:3/18:1)	[M-H]^−^
						PE (20:4/18:0)	[M-H]^−^
						PE (22:4/16:0)	[M-H]^−^
					5	PE (18:1/18:0)	[M + Na-2H]^−^
KID 1	740.75	740.5285	0.238	0.855	6	PE (18:2:18:1)	[M-H]^−^
						PE (18:3/18:0)	[M-H]^−^
						PE (20:3/16:0)	[M-H]^−^
					7	PC (18:3/16:0)	[M-CH_3_-H]^−^
					9	PE (18:0/16:0)	[M + Na-2H]^−^
						PE-NMe_2_ (16:0/16:0)	[M + Na-2H]^−^
LG 1	642.25	642.4875	0.314	0.807	—	*N/A*	*N/A*
LG 2	687.75	687.5363	0.278	0.895	4	PA (O-18:0/18:1)	[M-H]^−^
						PA (P-18:0/18:0)	[M-H]^−^
						PA (P-20:0/16:0)	[M-H]^−^
LG 3	818.75	818.7625	0.197	0.935	—	*N/A*	*N/A*
LIV 1	727.75	727.5286	0.233	0.855	0	PA (O-18:0/20:3) (OH)	[M-H]^−^
						PA (20:0/18:2)	[M-H]^−^
						PA (20:1/18:1)	[M-H]^−^
						PA (20:2/18:0)	[M-H]^−^
						PA (22:2/16:0)	[M-H]^−^
					5	PE (18:1/18:0)	[M-NH_4_]^−^
LIV 2	749.75	749.5130	0.266	0.967	0	PA (20:3/20:2)	[M-H]^−^
						PA (20:4/20:1)	[M-H]^−^
						PA (20:5/20:0)	[M-H]^−^
						PA (22:4/18:1)	[M-H]^−^
						PA (22:5/18:0)	[M-H]^−^
					3	PA (20:0/18:2)	[M + Na-2H]^−^
					5	PE (20:3/18:1)	[M-NH_4_]^−^
						PE (20:4/18:0)	[M-NH_4_]^−^
LIV 3	775.75	775.5306	0.239	0.960	2	PE (22:5/18:0)	[M-NH_4_]^−^
					3	PA (22:5/20:1)	[M-H]^−^
						PA (22:6/20:0)	[M-H]^−^
					4	MGDG (18:2/16:0)	[M + Na-2H]^−^
					6	PA (20:3/20:0)	[M + Na-2H]^−^
					8	MGDG (18:3/18:2)	[M-H]^−^
LIV 4	833.75	833.6292	0.280	0.772	2	PG (20:0/20:0)	[M-H]^−^
						PG (22:0/18:0)	[M-H]^−^

Table 2 identifies that most of the assigned brain lipid identifications are sphingolipid Cer species and not phospholipids as opposed to the other offal cuts. The predominant assignment of Cer species is somewhat concurrent with a recent New Zealand Wagyu study which identified abundant levels of sphingomyelins (SM) in brain compared to other offal tissues^30^. Another interesting observation within Table 2 is the lack of polyunsaturated chains assigned for the chosen brain ions. Such observations were also reported in a recent rat phospholipid study which suggested that brain is deficient in the number of long-chained polyunsaturated chains compared to heart, kidney and liver^31^. Although it is commonly perceived that brain lipid species, particularly phospholipids, are highly enriched with long-chained polyunsaturated chains, there may be evidence disputing such ideologies.

**Figure 3:**
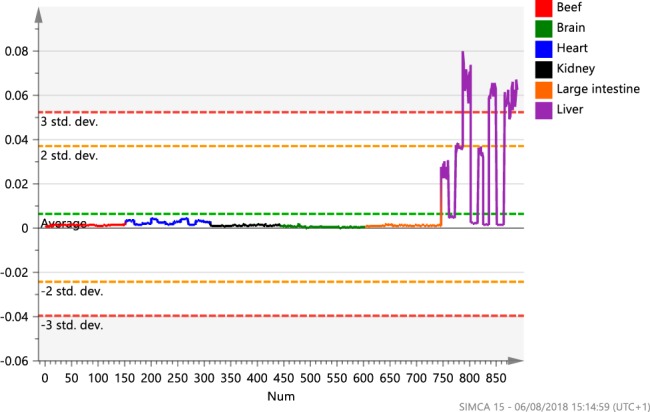
A trend plot of ion m/z 833.75 (MS −ve) (LIV 4) representing the abundance of the ion within each cut for all samples analysed by the REIMS technology. It demonstrates that the ion was only present in liver samples. However, the figure also clearly shows that the ion is not present within 4/11 (36%) of the liver samples analysed. Therefore, it cannot be considered a unique marker. Such observations maybe a result of sex, breed, geographic origin or feed.

Two potential unique markers were believed to have been identified within this study. BRN 5 was identified as GluCer (d18:24:1) and LIV 4 a mixture of PA (24:0/18:0), PG (20:0/20:0) and PG (22:0/18:0). However, further analysis identified that LIV 4 was not present in 4/11 (36%) liver samples as shown in the variable trend plot (Fig. 3). This may be a result of a multitude of variables such as sex, breed, age, geographic origin or, most likely, feed as liver was the only tissue that exhibited such ion variation. Thus, due to inconsistencies it cannot be referred to as a unique marker. Importantly, such scenarios demonstrate the need for large sample sets and their associated meta data when conducting metabolomics studies. With regards to BRN 5, it may be possible to specifically target it instead of scanning the full mass range to identify brain adulteration.

### Real-time adulteration of boiled beef burgers

**Figure 4:**
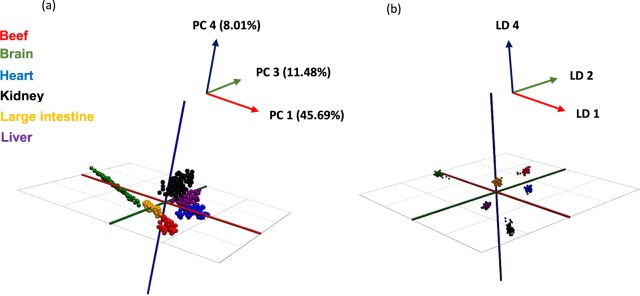
(**a**) PCA score plot and (**b**) LDA plot of REIMS-QTof MS spectral data (m/z 600–950) obtained from boiled beef muscle, brain, heart, kidney, large intestine and liver tissues negative ionisation and sensitivity mode. Each data point represents a single cut within the experiment of which there were 655 in total. 70 PCA components, 5 LDA components and a mass bin size of 0.5 Da were used. R^2^ = 0.975 and Q^2^ = 0.943 values were obtained for the PCA score plot.

A large proportion of commercially available products are readymade meals therefore, the applicability of the REIMS technology to cooked samples was assessed by boiling samples and adulterated burgers at 95 °C for five minutes. Like the raw study, a variety of mass ranges were tested when generating chemometric models, however, after thorough investigation it was deemed that the greatest separation was present within the mass range m/z 600–950 (Fig. 4). Utilising the recognition software, the detected adulteration levels for each adulterant were; brain (5–10%); heart (1–10%); kidney (1–5%); large intestine (1–10%) and liver (5–10%) with a full summary of the findings outlined in Supplementary S2. Compared to the raw tissue results, only burgers which had been adulterated with brain produced outlier classifications indicating that the cooking treatment diminished some of the spectral differences between the six tissues. The use of 70 PCA components compared to the 50 used for the raw tissue model also illustrates this. This may indicate that after ‘x’ amount of time the tissues could be indistinguishable. However, the results demonstrate that after some form of heat processing treatment, REIMS is still capable of detecting adulteration at acceptable levels and that specific offal cuts can still be identified. The focus for further studies should be on identifying whether one model can incorporate multiple aspects of cooking (grilling, frying, steaming) or if individual models are required for each cooking method.

## Experimental

### Sample collection and beef mincing

Beef muscle and offal tissues were obtained from highly trusted suppliers within Northern Ireland, Scotland and the Republic of Ireland. Fifteen samples of each different offal tissue were obtained, each one originating from a different carcass. Upon collection, all samples were stored at −80 °C. Beef muscle was minced using a Kenwood CH180 300 W mini chopper food processor prior to forming beef burgers (20 g) ready for REIMS analysis.

### Preparation of adulterated beef burgers

Adulterated beef burgers (20 g) with brain, heart, kidney, large intestine and liver were prepared by combining minced beef with offal samples in the mini chopper food processor until fully homogenised. Four of the fifteen samples for each offal cut were used to prepare the adulterated beef burgers with each individual offal sample being used to prepare four adulteration levels: 20, 10, 5 and 1%. This resulted in a total of sixteen adulterated burgers for each offal sample and eighty raw adulterated burgers in total. The remaining eleven samples of each offal adulterant were used to generate the chemometric training model. As none of the beef muscle or offal samples used to prepare the adulterated burgers were used in the chemometric training model building process, the samples could be considered a ‘blind’ test. The burgers were analysed to assess what adulteration levels were capable of detection using the REIMS technology.

### Boiling method and preparation of adulterated boiled beef burgers

To generate a chemometric model for boiled samples, minced beef and offal samples were heated in domestic boil-in-bags at 95 °C for five minutes. Samples were cooled to room temperature before being frozen at −80 °C. Adulterated beef burgers (20 g) were prepared in the same manner as set out previously then boiled as above. The samples used to prepare the raw and boiled adulterated beef burgers were identical. In total 160 adulterated burgers were analysed within this study.

### Instrumentation

A Waters REIMS source (Waters Corporation, Wilmslow, UK) was coupled to a Xevo G2-XS QTof mass spectrometer (Waters Corporation, Wilmslow, UK) which was operated in negative ion and sensitivity mode. Mass spectrometric data were acquired over the range m/z 50–1200 with a scan time of 0.5 s. The REIMS source was connected to a monopolar electrosurgical knife (Model PS01–63H, Hangzhou medstar technology Co, Ltd, Jiaxing City, China) through a 3 m long, 1 cm. diameter ultra-flexible tubing (evacuation/vent line). Electrosurgical dissection in all experiments was performed using an Erbe VIO 50 C generator (Erbe Medical UK Ltd, Leeds, UK). The generator was operated in ‘autocut’ mode with a power setting of 30 W. Samples were cut on the return electrode and a venturi gas jet pump evacuated the aerosol produced at the sample site and was introduced into the first vacuum stage via a stainless steel capillary where the aerosol droplets were mixed with aerosolised lockmass solution (see details below) and directed towards a heated kanthal coil (approx. 900 °C) that was operated at approximately 20 W. As specified risk materials (SRM) were analysed in this study, the necessary safety protocols were adhered to including all experimental cutting being conducted within a Contained Air Solutions (CAS) BioMat 1 class 1 microbiological safety cabinet which is fully compliant with EN 12469:2000.

A lockmass solution of Leucine Enkephalin (LeuEnk) (m/z 554.2615 [M-H^−^]) (0.1 ng / µL) in isopropanol (IPA) was infused using a Waters Acquity UPLC I-class system (Waters Corporation., Milford, MA, USA) at a continuous flow rate of 0.2 mL/min for accurate mass correction. Prior to analysis the time of flight (Tof) analyser was calibrated using 0.5 mM sodium formate solution (90% IPA) at a flow rate of 20 µL/min. Dependent on size, each tissue sample was cut 10–15 times for reproducibility with each cut lasting approximately 3–5 s. This enabled multiple locations on each tissue sample to be analysed. The delay between sampling and appearance of a signal was ≈2 s, with no carry-over effects visible between each cut and/or sample.

### Data pre-processing and chemometric analysis

Raw data generated by the mass spectrometer were pre-processed using a prototype abstract model builder software, AMX version 1.0.1581.0 (Waters Research Centre, Budapest, Hungary) that used standard Masslynx pre-processing algorithms (Waters). Data were background subtracted, lockmass corrected using LeuEnk (m/z 554.2615 [M-H^−^]) and normalised (Total Ion Count - TIC) before being exposed to multivariate analysis. All chemometric models were generated on a ‘per cut’ basis, i.e. each data point represents a single cut within the experiment and not a single sample. Additionally, all models were generated using the mass region m/z 600–950, a spectral intensity threshold of 2e^6^ counts and a bin width of 0.5 Da. PCA was used to reduce the dimensionality of the data prior to LDA analysis using the first 25 PCA components.

The processed PCA-LDA matrices generated within the AMX software were exported to SIMCA 15 (Umetrics, Umea, Sweden) allowing data to be exposed to further chemometric functions. All data were mean-centred, pareto scaled and grouped accordingly into the six classes before being analysed. R^2^ (cumulative), Q^2^ (cumulative) and Root Mean Squared Error of cross validation (RMSECV) were used to determine the validity of the models. R^2^ (cum) indicates the variation described by all components in the model and Q^2^ (cum) is a measure of how accurately the model can predict class membership. Permutation testing (200 permutations) was carried out to ensure the OPLS-DA models were not over-fitted to accommodate the training model but could not then predict Y (class identification) for new observations. Variable importance projections (VIP) was used to remove ions which did not have a significant baring on the dataset and then individual OPLS-DA models of each offal cut against the other five classes enabled S-plots to be created to identify the significant ions within the REIMS dataset. S-plot |p| and |p(corr)| values were used to validate ion selection for REIMS MS/MS analysis with the contribution of each variable (ion) to the variance of the observations being determined by the |p| value and the reliability of each variable for group separation identified by the |p(corr)| value. The REIMS technology was used to generate the MS/MS spectra as our goal was to identify the lipids generated with the thermal degradation through the REIMS method. To determine the appropriate collision energy (CE) for fragmentation, experiments for each ion were conducted starting at CE = 0 V and increased incrementally by 5 V until fragmentation was achieved. Putatively annotated identifications were assigned by searching peak m/z values in the METLIN metabolite database and LIPID MAPS with a threshold of 10 ppm.

### Real-time classification of samples

The PCA-LDA models generated within the AMX software were exported to a prototype recognition software enabling raw spectral data to be simultaneously acquired and classified. The time taken to cut a sample and determine classification was near-instantaneous (2 seconds). A standard deviation of 20σ was used to assign class identification. If a sample was outside the standard deviation range of 20σ for all classes, then an outlier classification would be assigned. Sample classifications were assigned after every second to provide the greatest possibility of detecting offal adulteration at the lower levels of adulteration.

## Conclusions

The study of minced beef adulteration with offal products has been reported for the first time using a mass spectrometric platform. The ambient nature of the REIMS technology enabled samples to be analysed and classified near-instantaneously without the need for any form of sample preparation whilst the coupling with HRMS allowed unique or significant markers to be identified. Specific offal identifications were obtained for both raw and boiled adulterated samples, an aspect which totally eludes DNA studies and has been identified as a major weakness for multiple vibrational spectroscopic studies, whilst the assignment of outliers was due to the analysis of ‘hybrid’ beef-adulterant spectra. Adulteration levels ranging from 1–10% adulteration were detectable for both raw and boiled burgers. Although there is a plethora of analytical techniques capable of detecting meat adulteration at lower levels, the levels observed within this study are more than sufficient to detect commercial adulteration perpetrated by criminals for economic gains. Chemometric analysis identified a wide range of lipid species responsible for separation within the raw samples whilst it appears that heat treatment cooking methods may nullify the amount of spectrometric variation that exists between the different tissue types. In conclusion, REIMS could provide a paradigm shift across many authenticity applications by providing real-time, specific offal identifications with the potential to identify unique or significant markers. In future it may be that vibrational spectroscopic platforms are used as a quick non-destructive screening method and then REIMS used as a confirmatory technique to identify the specific adulterant, whether that be an untargeted or targeted approach. Such two-tier approaches have already proved successful in detecting dried oregano adulteration^28,29^.

## Supplementary information

Supplementary information
