# Supplementary material for: Rapid detection and specific identification of offals within minced beef samples utilising ambient mass spectrometry
Source: Sci Rep. 2019 Apr 18;9:6295. doi: 10.1038/s41598-019-42796-5 (PMC6472348; doi:10.1038/s41598-019-42796-5)
Supplement: Supplementary file 1 — Supplementary information [file 41598_2019_42796_MOESM1_ESM.docx]

**Rapid detection and specific identification of offal’s within minced beef samples utilising ambient mass spectrometry -supplementary information**

Connor Black,^1*^ Olivier P. Chevallier,^1,2^ Kevin M. Cooper,^1^ Simon A. Haughey,^1^ Julia Balog,^3,4^ Zoltan Takats,^1,4^ Christopher T. Elliott,^1^ Christophe Cavin^5^

*^1^ Institute for Global Food Security, Advanced ASSET Centre, School of Biological Sciences, Queen’s University Belfast, 18-30 Malone Road, Belfast, United Kingdom*

*^2^ Mass Spectrometry Core Technology Unit, Queen’s University Belfast, United Kingdom*

*^3^ Waters Research Centre, 7 Zahony Street, Budapest, Hungary*

*^4^ Imperial College London, South Kensington Campus, Sir Alexander Fleming Building, London, United Kingdom*

*^5^ Nestlé Research, Vers-chez-les Blanc, CH-1000, Lausanne 26, Switzerland*

******corresponding author email address – connor.black@qub.ac.uk*

S1 – Table showing the observed identifications for the raw adulterated burgers when analysed using the REIMS technology. Specific offal identifications were assigned as well as outlier classifications due to the analysis of beef-adulterant ‘hybrid’ spectra at a specific scan time

S2 – Table showing the observed identifications for the boiled (95^o^C for 5 minutes) adulterated burgers when analysed using the REIMS technology. Specific offal identifications were assigned as well as outlier classifications due to the analysis of beef-adulterant ‘hybrid’ spectra at a specific scan time
